# Research progress of antibody-drug conjugates in the treatment of endometrial cancer

**DOI:** 10.3389/fonc.2026.1706826

**Published:** 2026-04-27

**Authors:** Yuying Jiang, Qin Yao

**Affiliations:** Department of Obstetrics and Gynecology, The Affiliated Hospital of Qingdao University, Qingdao, China

**Keywords:** antibody-drug conjugates, combination therapy, endometrial cancer, gynecological tumor, research progress

## Abstract

As a significant threat to women’s health worldwide, the incidence of endometrial cancer is rising annually, and current treatment options have limited effectiveness for advanced-stage patients. The use of antibody-drug conjugates (ADCs) in the treatment of endometrial cancer shows great potential. ADCs achieve selective killing of tumor cells through the combination of monoclonal antibodies, linkers, and cytotoxic drugs, while reducing damage to normal tissues. ADCs targeting Trop-2, HER2, and FRα have demonstrated notable efficacy in clinical trials, such as Sacituzumab Govitecan (SG) and Trastuzumab Deruxtecan (T-DXd), which have shown high objective response rates in Trop-2 and HER2-positive patients, respectively. Furthermore, the combination of ADCs with other treatment modalities (such as chemotherapy and immunotherapy) further enhances efficacy. However, ADCs still face challenges regarding resistance and safety, and future efforts must focus on optimizing designs and exploring more emerging targets to achieve the goals of precision medicine. This article provides a focused narrative overview of recent advances in ADCs for the treatment of endometrial cancer, with a primary focus on key targets including Trop-2, HER2, FRα, and Nectin-4.

## 1 Introduction

### 1.1 Current status of endometrial cancer

Endometrial cancer, a common malignant epithelial tumor originating in the endometrium, characterized by abnormal uterine bleeding as its typical clinical manifestation, posing a major threat to women’s health worldwide. According to World Health Organization (WHO) data, the age-standardized incidence of this disease in developed countries continued to rise from 2003 to 2015 (**Supplementary Figure 1**), ranking first among gynecological malignancies (1). Bray et al. based on 2022 global cancer statistics (2), further indicated that it ranks second in incidence among gynecological malignancies in 185 countries (accounting for 8.4%, second only to cervical cancer) and third in mortality (**Supplementary File 1**). In recent years, there has been a notable improvement in the prognosis for endometrial cancer patients as a result of advancements in treatment methods, including surgery, chemotherapy, radiotherapy, and immunotherapy. However, these existing treatment methods are still inadequate for patients with advanced, refractory, recurrent, and metastatic disease. Exploration of novel treatment strategies is imperative to enhance efficacy and patient prognosis (3).

### 1.2 Concept and current status of antibody-drug conjugates

Antibody-drug conjugates (ADCs), as a representative of precision-targeted therapy, have become not only the focus of numerous clinical studies but also a key component in clinical practice (4). ADCs consist of monoclonal antibodies, a linker, and a small-molecule cytotoxic drug (5). The core mechanism is based on the “targeted delivery-highly effective killing” model, which selectively delivers cytotoxic drugs to tumor cells to achieve precision therapy. Compared with free drugs, ADCs exhibit higher tumor selectivity (6), while demonstrating limited off-target toxicity and potential bystander effects (7). However, a careful risk-benefit assessment is required, as clinically common target/non-target effects and ADCs-induced systemic toxicities such as hematologic toxicity, gastrointestinal reactions, and neurotoxicity remain prevalent (8). As of October 2025, 15 ADCs had been approved by the Food and Drug Administration (FDA) and were being used extensively in the treatment of hematological and solid tumors (**Table 1**). At present, two ADCs have been approved by the FDA for the treatment of gynecological malignancies: Tisotumab Vedotin (TV) for cervical cancer and Mirvetuximab Soravtansine (MIRV) for ovarian cancer (9).

**Table 1:** 15 FDA-approved ADCs as of October 2025.

Drug name	Target	Indications	FDA first approval date	Drug status
Gemtuzumab Ozogamicin	CD33	CD33-positive acute myeloid leukemia	May 2000	Withdrawn in November 2011 Re-approval in September 2017
Brentuximab Vedotin	CD30	Hodgkin lymphoma	August 2011	Active
Trastuzumab Emtansine	HER2	HER2-positive metastatic breast cancer	February 2013	Active
Inotuzumab Ozogamicin	CD22	Relapsed/refractory B-cell acute lymphoblastic leukemia	August 2017	Active
Moxetumomab Pasudotox	CD22	Hairy cell leukemia	September 2018	Withdrawn in November 2022
Polatuzumab Vedotin	CD79b	Diffuse large B-cell lymphoma	June 2019	Active
Enfortumab Vedotin	Nectin-4	Advanced urothelial carcinoma	December 2019	Active
Trastuzumab Deruxtecan	HER2	HER2-positive metastatic breast cancer	December 2019	Active
Sacituzumab Govitecan	Trop-2	Triple negative breast cancer	April 2020	Active
Belantamab Mafodotin	BCMA	Relapsed/refractory multiple myeloma	August 2020	Withdrawn in November 2022 Re-approval in October 2025
Loncastuximab Tesirine	CD19	Relapsed/refractory large B-cell lymphoma	April 2021	Active
Tisotumab Vedotin	TF	Recurrent/metastatic cervical cancer	September 2021	Active
Mirvetuximab Soravtansine	FRα	FOLR1-positive, platinum-resistant epithelial ovarian, fallopian tube, or primary peritoneal Cancer	November 2022	Active
Datopotamab Deruxtecan	Trop-2	Unresectable/metastatic, HR-positive, HER2-negative breast cancer	January 2025	Active
Telisotuzumab Vedotin	c-Met	Advanced/metastatic non-squamous non-small cell lung cancer with high c-Met protein overexpression	May 2025	Active

### 1.3 Research status of ADCs in the treatment of endometrial cancer

Although no ADCs have been formally approved for the treatment of endometrial cancer, their successful applications in other malignancies have laid the groundwork for exploring their potential in this disease. Additionally, multiple clinical trials of ADCs have demonstrated encouraging efficacy, such as Sacituzumab Govitecan (SG) and Trastuzumab Deruxtecan (T-DXd), which showed high objective response rates (ORR) in Trop-2- and HER2-positive EC patients, respectively (10, 11). Currently, comprehensive summaries of ADCs for endometrial cancer treatment remain limited. Existing studies focus predominantly on clinical trials of individual ADCs or pan-cancer efficacy evaluations, rather than syntheses specific to endometrial cancer. This review aims to critically synthesize the current state of ADCs research in endometrial cancer treatment, including target selection, clinical trial outcomes, and combination therapy strategies, while also discussing the limitations of existing studies and future directions to inform clinical practice and subsequent research. Specifically, this review will sequentially evaluate the performance of ADCs targeting key targets (Trop-2, HER2, FOLR1, and Nectin-4) in clinical endometrial cancer trials (**Table 2**), analyze synergistic effects of ADCs combinations with chemotherapy or immunotherapy, and objectively assess ongoing challenges in ADCs development, such as off-target toxicity and therapeutic resistance.

**Table 2:** Summary of clinical trials for ADCs targeting endometrial cancer as of October 2025.

ADCs	Target	Trial phase	Clinical trial number	Clinical results	Study participants	References
Sacituzumab Govitecan	Trop-2	Phase I/II	NCT01631552	ORR 22.2%, mPFS:3.2 months, mOS:11.9 months.	Patients with advanced epithelial cancers	(12)
Sacituzumab Govitecan	Trop-2	Phase II	NCT03964727	ORR 22%, CBR 32%, mDOR 8.8 months, mPFS:4.8 months.	Patients with metastatic solid tumors	(11)
Trastuzumab Deruxtecan	HER2	Phase II	NCT04482309	In pooled pan-tumor patients: ORR 37.1%, mDOR 11.3 months, mPFS was 6.9 months, mOS 13.4 months. Subgroup: In patients with central HER2 IHC 3+ expression: ORR 61.3%, mDOR 22.1 months, mPFS 11.9 months, mOS 21.1 months; In endometrial cancer patients: ORR 57.5%, mPFS was 11.1 months, mOS 26.0 months; In endometrial cancer patients with central HER2 IHC 3+ expression: ORR 84.6%.	HER2-expressing (immunohistochemistry [IHC] 3+/2+ by local or central testing) locally advanced or metastatic disease after ≥1 systemic treatment or without alternative treatments	(13, 14)
Trastuzumab Deruxtecan	HER2	Phase II	NCCH1615	ORR in HER2-high and HER2-low groups were 54.5% and 70.0%. The mPFS and mOS in HER2-high and HER2-low groups were 6.2 and 13.3 months and 6.7 months and not reached.	Patients with uterine carcinosarcoma (UCS) expressing HER2	(15)
DB-1303	HER2	Phase I/II	NCT05150691	ORR 58.8% and DCR 94.1% in 17 evaluable patients. Among the subjects who received the 7.0 mg/kg dose, the ORR was 50.0%, while in the 8.0 mg/kg group, the ORR increased to 61.5%.	Patients with advanced endometrial cancer	(4, 9)
Mirvetuximab Soravtansine	FRα	Phase II	NCT03835819	ORR 37.5%, SD 31.3%.	FRα-positive recurrent/persistent endometrial cancer	(16, 17)

All clinical trial basic information is retrieved from ClinicalTrials.gov, while efficacy outcomes are derived from the latest published references. ORR: objective response rates; mPFS: median progression-free survival; mOS: median overall survival; CBR: clinical benefit rate; mDOR: median duration of response; DCR: disease control rate; SD: stable disease; References are listed at the end of the review.

## 2 Research on ADCs targeting key targets in endometrial cancer treatment

### 2.1 Trop-2-targeted ADCs

Trop-2 is a transmembrane glycoprotein with multifaceted roles in tumor cell proliferation, invasion, and metastasis, involving the regulation of multiple cellular signaling pathways (18, 19). In the GEPIA2 and GENT2 databases, it exhibits significantly higher expression in endometrial cancer tumor tissues compared to normal tissues, with higher expression in endometrioid subtypes than in serous carcinomas (**Supplementary Figure 2**). As a biomarker closely related to endometrial cancer, Trop-2 also shows great potential in the diagnosis of endometrial cancer and the prediction of poor prognosis (20). Long et al. studies have identified a positive correlation between Trop-2 expression levels and the severity of endometrial lesions. Patients diagnosed with endometrial cancer who exhibit high levels of Trop-2 expression have been observed to have significantly lower survival rates (21). Furthermore, the expression level of Trop-2 in endometrial cancer is closely related to tumor grading and cervical involvement (18), as well as negatively correlated with tumor differentiation (22). These findings provide new ideas and methods for the personalized treatment of endometrial cancer with ADCs targeting Trop-2.

Sacituzumab Govitecan (SG) is the first FDA-approved ADC targeting Trop-2, which has shown significant efficacy in the treatment of triple-negative breast cancer. Currently, several clinical trials of SG for the treatment of endometrial cancer are actively underway (11). A study (NCT01631552) targeting patients with metastatic endometrial cancer who had previously received at least one systemic platinum-based chemotherapy demonstrated that the ORR of patients treated with SG reached 22.2%, with a median progression-free survival (PFS) of 3.2 months and a median overall survival (OS) of 11.9 months (12). SG has demonstrated encouraging antitumor activity in heavily pretreated endometrial cancer patients, and its application value in multiply relapsed and advanced endometrial cancer is currently being further evaluated through clinical trials (9). In addition, Datopotamab deruxtecan is a novel FDA-approved Trop-2-targeted ADC that has demonstrated remarkable preclinical activity against poorly differentiated endometrial cancer cell lines overexpressing Trop-2 in *in vitro* studies (23). With the in-depth exploration of the mechanism of action of Trop-2 in endometrial cancer, as well as the research and development and clinical application of more Trop-2 ADCs, its application prospects in the field of endometrial cancer diagnosis and treatment will be even broader.

### 2.2 HER2-targeted ADCs

HER2 is a tyrosine kinase receptor membrane glycoprotein encoded by ERBB2, which is overexpressed in many solid tumors, including breast cancer, gastric cancer, and non-small cell lung cancer (24, 25). Research has demonstrated that HER2 has been identified as a potential therapeutic target in endometrial cancer (26). Approximately 30% to 35% of uterine serous carcinoma cases exhibit ERBB2 gene amplification or overexpression, while this rate ranges from 15% to 20% in uterine carcinosarcoma (27). This phenomenon is closely related to increased tumor invasiveness and poor patient prognosis (4). The targeting of this pathway has yielded encouraging results in the context of clinical trials involving several ADCs. At present, two ADCs targeting HER2 have been approved by the FDA: Trastuzumab Emtansine, Trastuzumab Deruxtecan (T-DXd). Despite the absence of formal approval for the use of these ADCs in the treatment of endometrial cancer, their efficacy has been demonstrated in clinical trials.

In the Phase II DESTINY-PanTumor02 trial (NCT04482309), a key trial evaluating the application of T-DXd in locally advanced or metastatic tumors, a cohort of 40 patients with endometrial cancer was enrolled. The investigator-assessed ORR of this cohort reached 57.5%. Of these patients, 13 were confirmed by central laboratory testing to have HER2 immunohistochemical (IHC) 3+ expression, and the ORR in this subgroup further increased to 84.6% (95% confidence interval [CI]: 54.6-98.1). Additionally, the median PFS and OS among all endometrial cancer patients were 11.1 months and 26.0 months, respectively. These data clearly demonstrate the significant therapeutic potential of T-DXd in HER2-positive endometrial cancer. (13, 14). The STATICE trial, a phase II single-arm study, evaluated T-DXd in patients with advanced or recurrent uterine carcinosarcoma (UCS, a high-grade endometrial cancer) who had received prior chemotherapy and exhibited HER2 expression (IHC ≥1+). The trial demonstrated notable antitumor activity, with an ORR of 54.5% (95% CI, 32.2–75.6) in the HER2-high group (IHC ≥2+) and 70.0% (95% CI, 34.8–93.3) in the HER2-low group (IHC 1+) by central review. The median PFS was 6.2 months in the HER2-high group and 6.7 months in the HER2-low group. These results are encouraging, given the historically poor prognosis of recurrent UCS (15). In consideration of the aforementioned clinical benefits, the FDA granted accelerated approval for T-DXd in adult patients with unresectable or metastatic HER2-positive (IHC 3+) solid tumors who have previously received systemic therapy and lack satisfactory alternative treatment options on April 5, 2024. (28). This development signifies the significant potential of T-DXd in the management of advanced recurrent HER2-positive endometrial cancer. In addition, DB-1303, a novel ADC targeting HER2, demonstrated significant efficacy in a Phase I/II clinical trial (NCT05150691) for the treatment of advanced endometrial cancer, with the ORR of 58.8% and the disease control rate of 94.1% in 17 evaluable patients. Among the subjects who received the 7.0 mg/kg dose, the ORR was 50.0%, while in the 8.0 mg/kg group, the ORR increased to 61.5% (4, 9). It is evident from the data presented that the FDA has granted fast track designation to DB-1303 for the treatment of HER2-positive advanced and metastatic endometrial cancer (29). This development indicates that DB-1303 represents a novel therapeutic option for patients with this particular indication, following the introduction of T-DXd. ADC targeted therapy for patients with HER2-positive endometrial cancer is the subject of ongoing refinement, with the potential that it may serve as a supplement or alternative to standard chemotherapy for this cohort in the future, pending further comparative clinical data.

### 2.3 FRα-targeted ADCs

FRα is expressed at low levels in normal tissues, but is significantly overexpressed on the surface of various epithelial tumor cells, including those responsible for ovarian, endometrial, cervical, breast, and lung cancer (30). This tumor-selective expression characteristic renders it a popular target for the development of new anticancer drugs and has attracted much attention in the field of targeted therapy (31). MIRV is an FRα-targeted ADC that has been approved by the FDA for the treatment of FRα-positive, platinum-resistant epithelial ovarian cancer (32) and has demonstrated significant antitumor activity in multiple clinical trials. A phase II clinical trial was conducted by Rebecca et al. in patients with FRα-positive recurrent/persistent endometrial cancer (16). The combination of MIRV and the PD-1 inhibitor pembrolizumab resulted in objective remission in 37.5% of patients, while stable disease was observed in an additional 31.3% (17).

STRO-002, a novel ADC targeting FRα, has demonstrated significant antitumor activity in preclinical studies: Li et al. utilized an animal model to verify that a solitary administration of STRO-002 potently suppresses the proliferation of FRα-positive neoplasms (33). Accordingly, the pharmaceutical compound is presently undergoing Phase I clinical trials (NCT03748186) in patients diagnosed with advanced epithelial ovarian cancer and endometrial cancer. The primary objective of these trials is to evaluate the efficacy and safety of the compound in populations exhibiting high FRα expression. Concurrently, a novel generation of FRα-targeted ADC, such as Rina-S (NCT05579366), has been initiated for early clinical studies targeting advanced solid tumors (including endometrial cancer) (30). To date, these trials have only published preliminary safety and efficacy data. For instance, Rina-S demonstrated good tolerability across the dose range of 60 mg/m² to 120 mg/m², with the maximum tolerated dose of 140 mg/m² still under evaluation. Among 21 patients with evaluable efficacy in ovarian and endometrial cancer, 1 achieved an unconfirmed complete response, 7 had partial responses, and 9 remained stable. The dose escalation phase of these drugs is still ongoing, indicating that FRα-targeted therapy holds significant potential in the field of gynecological oncology.

### 2.4 Other-targeted ADCs

In addition to Trop-2, HER2, and FRα, researchers are also exploring the potential application of targets such as Nectin-4 in the treatment of endometrial cancer. In the study by Chang et al. (34), a comparative analysis of 320 endometrial cancer tissue samples and 87 non-adjacent normal endometrial tissue samples revealed that Nectin-4 expression was significantly higher in cancer tissues (P<0.001), a difference that provides important reference value for endometrial cancer diagnosis. Further studies showed that high Nectin-4 expression is closely associated with poor prognosis in patients with DNA mismatch repair deficiency subtypes; patients with MSH2 deficiency and high Nectin-4 expression had significantly shorter PFS (Hazard Ratio [HR]=9.025, p=0.0286) compared with the low Nectin-4 expression group. Notably, this study did not involve efficacy analysis of ADCs, so the potential of Nectin-4 as a predictive biomarker for ADCs efficacy requires validation in specific clinical trials. Currently, Nectin-4-targeted ADCs are being explored in relevant fields. Enfortumab Vedotin has not been approved for gynecological malignancies but has shown positive efficacy in urothelial carcinoma treatment, and a Phase II clinical trial (NCT07139977) for advanced endometrial cancer is ongoing. Additionally, domestically developed Nectin-4-targeted ADCs (e.g., 9MW2821, SHR-A2102) are in clinical trials and are expected to provide new options for endometrial cancer treatment (35, 36).

Furthermore, the GEPIA2 database, as an initial bio-informatics screening platform, revealed that the expression levels of target genes such as ERBB3, NaPi2b, B7-H4, CD74, and MUC1 are significantly higher in endometrial cancer tissue than in normal tissue (37). Notably, differential expression identified by GEPIA2 only generates hypotheses, and multi-layered validation (e.g., confirmation of protein expression, tumor-specific localization, definition of a safety window, and functional validation) is still required to confirm whether these potential targets are suitable for ADCs development. In the future, with the advancement of more targeted ADCs research and development and clinical trials, the treatment of endometrial cancer will undergo even greater changes.

## 3 Combination use of ADCs with other treatments

In order to enhance the efficacy of ADCs, researchers have initiated the exploration of combining them with other treatment modalities, including chemotherapy, targeted therapy, and immunotherapy. The combination of ADCs with chemotherapy drugs has been shown to enhance the cytotoxic synergistic effects and demonstrate synergistic activity in tumor killing. The combination of ADCs with anti-angiogenic drugs has been demonstrated to promote tumor vascular normalization and significantly improve the penetration and distribution of ADCs in solid tumors (38). A Phase 1b study conducted by Richardson et al. directly validated the clinical value of the combination regimen. The study utilized a triple combination of MIRV, carboplatin (chemotherapy), and bevacizumab (an anti-angiogenic drug) to treat patients with platinum-sensitive, FRα-positive recurrent ovarian cancer. The findings demonstrated that this multi-mechanism, synergistic triple regimen exhibited not only elevated antitumor activity (with a significantly superior ORR compared to conventional regimens), but also exhibited good tolerability in patients, with no uncontrollable adverse reactions (39).

In recent years, the role of immune checkpoint inhibitors (ICIs) in the treatment of gynecological malignancies has continued to grow. Research has demonstrated that ADCs not only directly induce tumor cell apoptosis but also activate the host immune system by inducing tumor cells to release antigens. When used in combination with ICIs, they have been shown to enhance the body’s antitumor immune response and promote the efficacy of immunotherapy against tumors (40, 41). In the contemporary clinical context, the diagnosis and treatment of endometrial cancer frequently involve a combination of targeted therapy and immunotherapy. The rationale underpinning this approach is to enhance the anti-tumor efficacy through the generation of synergistic effects. For example, the KEYNOTE-775 trial confirmed that Pembrolizumab combined with Lenvatinib significantly prolonged OS (median OS: 18.3 vs. 11.4 months; HR = 0.62) and PFS (median PFS: 7.2 vs. 3.8 months; HR = 0.56) in patients with advanced endometrial cancer compared to traditional chemotherapy, with statistically significant differences (p<0.001) (42). The benefit was observed in both the overall population and specifically in the mismatch repair-proficient subgroup, where the median OS was 17.4 vs. 12.0 months (HR = 0.68) and median PFS was 6.6 vs. 3.8 months (HR = 0.60). Although the trial was not powered to show significance in the mismatch repair-deficient subgroup, clinically meaningful improvements were also observed. This combination strategy has led to further exploration of the combination of ADCs and ICIs, with the potential to overcome the limitations of single-agent therapy and further improve treatment outcomes.

## 4 Challenges and prospects for ADCs in the treatment of endometrial cancer

In the treatment of endometrial cancer, despite the evident potential of ADCs (an innovative combination of the macromolecular targeting property of antibodies and the killing effect of small molecule toxins), issues concerning drug resistance and safety remain significant barriers to their widespread utilization. The structural characteristics of the antibody, the selection of targets, the type of toxin, and the type of linker are the fundamental factors determining the capacity of ADCs to overcome these barriers and achieve clinical success.

At present, all common ADCs are based on traditional monoclonal antibody structures, and only a small number of ADCs contain bispecific antibodies. These bispecific antibody-drug couplings represent a novel type of ADC, with the capability of targeting two independent antigens concurrently. This capacity is anticipated to address the limitations of conventional ADCs, namely their low internalization efficiency, off-target toxicity, and vulnerability to drug resistance. It is noteworthy that more than a dozen bispecific ADCs have already entered the late stage of development, including M1231, which targets EGFR/MUC1; JSKN016, which targets HER3/Trop-2; BL-B01D1, which targets HER3/EGFR; and AK146D1, which targets Trop-2/Nectin4. In addition, dozens more are in clinical development. Nevertheless, the advancement of clinical applications of bispecific ADCs has been gradual, primarily constrained by limitations in production and manufacturing processes, including elevated production costs and intricate manufacturing procedures. These manufacturing challenges must be addressed in the future to facilitate the wider use of bispecific ADCs in clinical practice (43, 44). Secondly, in terms of target selection, we have observed that most current ADCs are designed around classic targets such as HER2, Trop-2, FOLR1, and Nectin-4. This highlights the limitations of ADCs in this area. In the future, we should accelerate research into emerging targets. We should focus on targets that are highly expressed in endometrial cancer cells but not or minimally expressed in normal tissues. Such targets could significantly enhance the accuracy with which ADCs target cancer cells, while reducing the systemic toxicity resulting from off-target effects. Secondly, we should focus on tumor-related targets that are highly expressed in different pathological subtypes of endometrial cancer, such as endometrioid carcinoma, serous carcinoma, and clear cell carcinoma. Due to significant differences in molecular mechanisms between subtypes, targeted selection can encompass a broader range of patients and facilitate personalized treatment development. We hope that, through this strategy, we will be able to identify more ADCs targets that have greater potential for clinical translation, thereby providing a scientific basis for subsequent drug development that is more closely aligned with the pathological characteristics of endometrial cancer (37).

Additionally, many ADCs have failed during clinical development due to excessive toxicity. The primary manifestation of this toxicity largely depends on the type of payload (45). The linker also plays a crucial role in the efficacy of ADCs. Non-cleavable linkers rely on lysosomal degradation and are more stable in plasma, thus avoiding the premature release of toxins into the bloodstream and subsequent adverse systemic reactions. However, their complete dependence on intracellular lysosomal proteolysis means inefficient toxin release may occur if lysosomal function is impaired. Cleavable linkers are categorized by activation location: peptide-based linkers (e.g., Val-Cit) release toxins intracellularly via lysosomal cathepsins, while acid-sensitive (hydrazone) or matrix metalloproteinase-sensitive linkers respond to extracellular tumor microenvironment conditions (low pH or matrix metalloproteinases). Preclinical and clinical evidence shows their sensitivity can lead to off-target release, which increases systemic exposure to cytotoxic agents, and established exposure-adverse event relationships link this systemic exposure to common toxicities such as myelosuppression and gastrointestinal mucosal damage. Therefore, ADCs’ design must strike a balance between plasma stability and responsiveness to the tumor microenvironment.

## 5 Conclusion

Thanks to their unique properties, antibody-drug conjugates offer new hope in the treatment of endometrial cancer. ADCs targeting Trop-2, HER2, and FRα have shown promise, and combination treatment strategies involving chemotherapy and immunotherapy have further enhanced the anti-tumor effect. However, ADCs still face challenges in terms of drug resistance and safety. The design of linkers and the selection of payloads in particular need to be optimized. As research on bispecific ADCs and emerging targets progresses, ADCs are expected to play a key role in personalized treatment for endometrial cancer, offering patients more options.

## Supplementary material

Supplementary File 1 Incidence (cases, age-standardized rate, cumulative risk) and mortality (deaths, age-standardized rate, cumulative risk) of five female cancers in 185 countries in 2022.
