# Supplementary figures and images for: Research progress of antibody-drug conjugates in the treatment of endometrial cancer
Source: Front Oncol. 2026 Apr 27;16:1706826. doi: 10.3389/fonc.2026.1706826 (PMC13158215; doi:10.3389/fonc.2026.1706826)

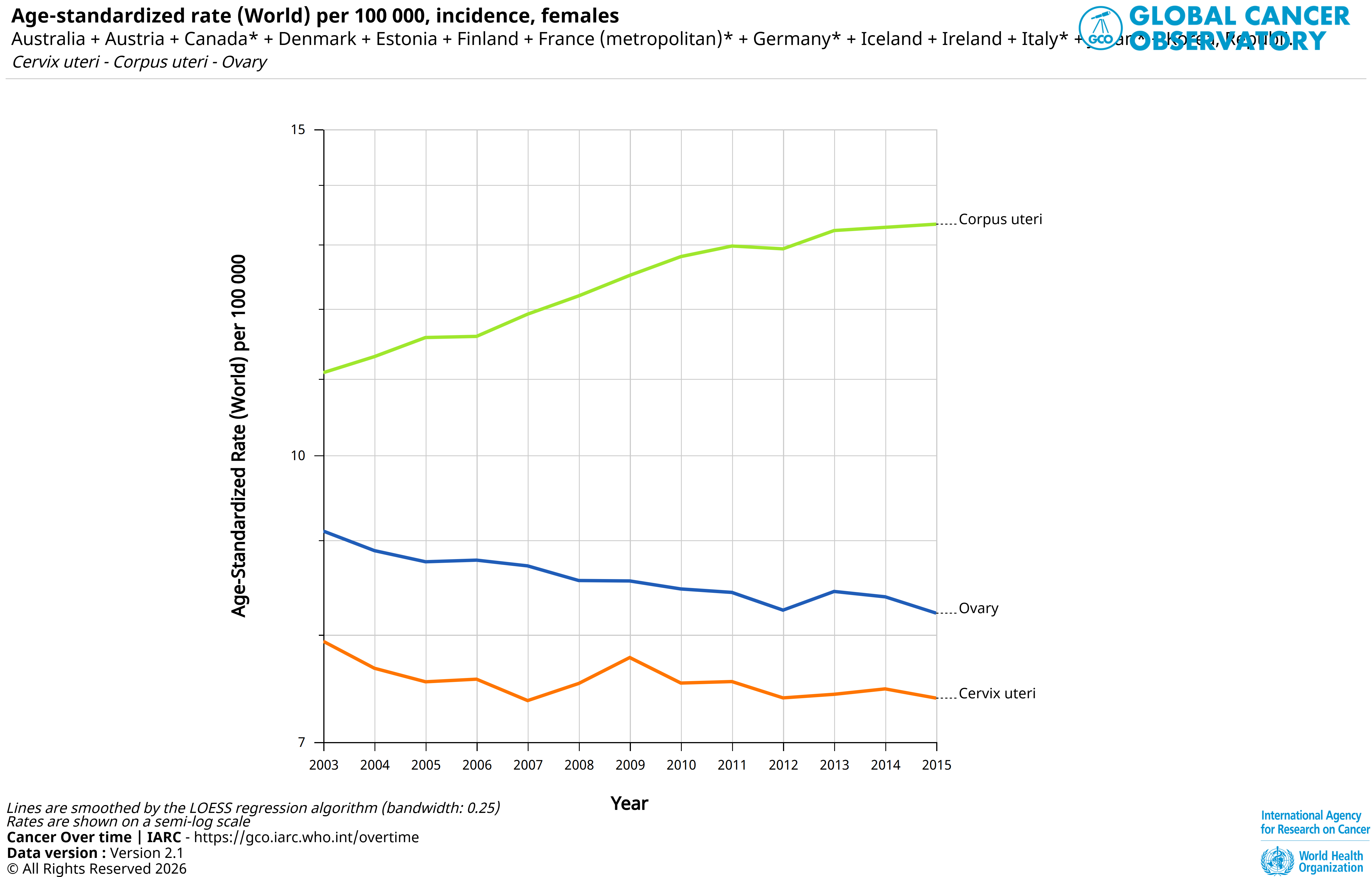

Supplement: Supplementary file 2 [file Image1.png]

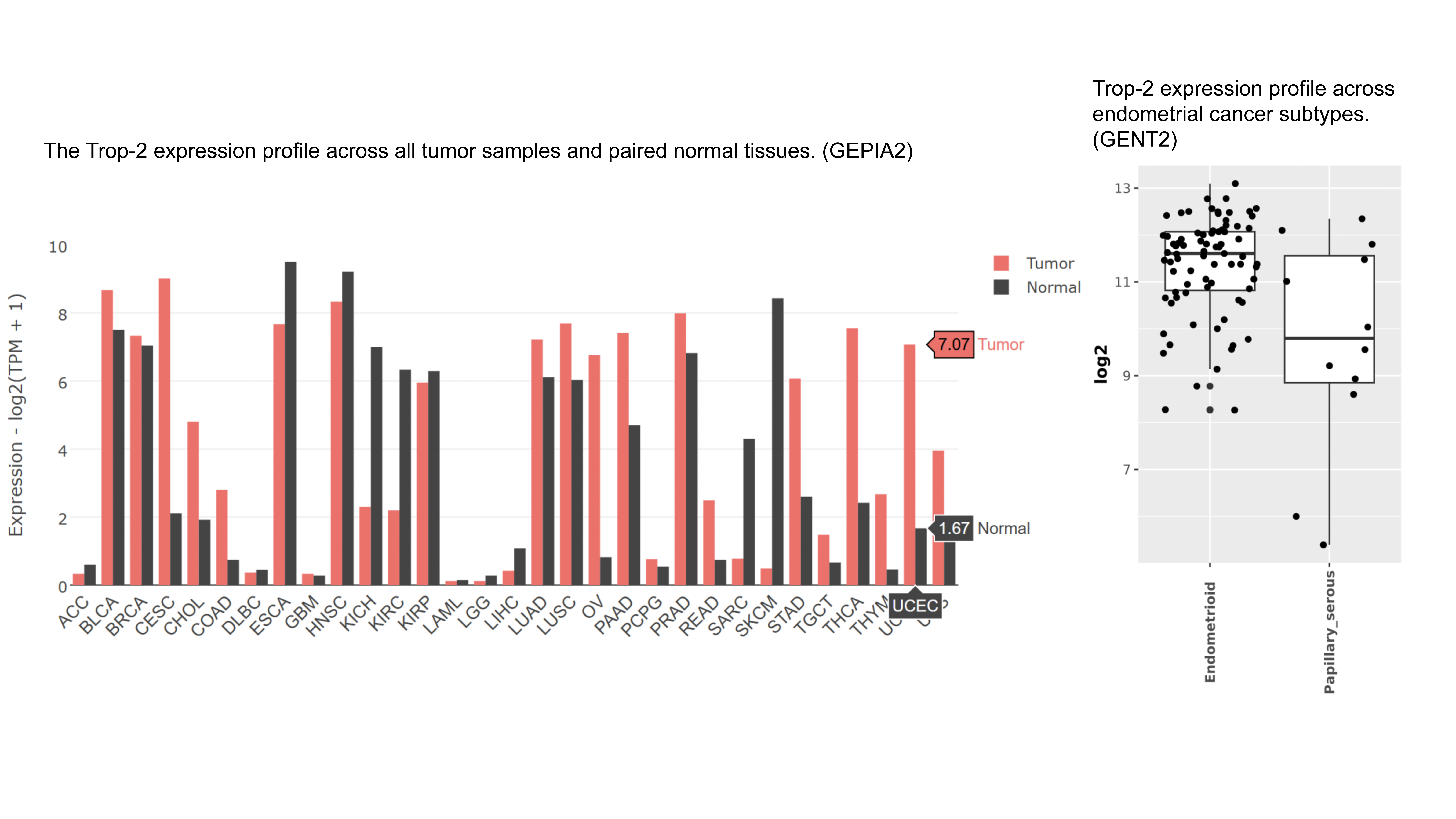

Supplement: Supplementary file 3 [file Image2.png]
